# Efficacy of Ozurdex implants as second-line therapy for non-responders to anti-VEGF in retinal vein occlusion-associated macular edema: a retrospective cohort study

**DOI:** 10.1007/s11845-025-03881-z

**Published:** 2025-01-24

**Authors:** Mahmoud Eissa, Dimitrios Kalogeropoulos, William Evans, Rashi Arora, Andrew John Lotery

**Affiliations:** 1 Salisbury Eye Unit, Salisbury District Hospital, Salisbury, UK; 2 Southampton Eye Unit, University Hospital Southampton, Southampton, UK; 3 Clinical and Experimental Sciences, Faculty of Medicine, South Lab and Path Block, Level D, University of Southampton, University Hospital Southampton, Mailpoint 806, Southampton, SO16 6YD UK

**Keywords:** Anti-VEGF therapy, Central retinal thickness, Macular edema, Ozurdex, Retinal vein occlusion

## Abstract

**Purpose:**

Retinal vein occlusion (RVO) is a prevalent retinal vascular disorder characterized by retinal haemorrhage, neovascularization, and macular edema This study aimed to assess the structural and functional effects of intravitreal implant (Ozurdex) treatment as a second-line for RVO-associated macular edema in patients who did not respond to first-line anti-VEGF therapy.

**Materials and methods:**

We conducted a retrospective observational cohort study using electronic health records of RVO patients at Salisbury District Hospital between January 2014 and December 2019. Inclusion criteria included patients diagnosed with central or branch RVO. Patients underwent ophthalmic evaluations at baseline, including central retinal thickness (CRT) and best-corrected visual acuity (BCVA) assessments. Statistical analysis was performed using IBM SPSS Statistics, employing various tests to determine significance.

**Results:**

Sixteen eyes of 16 White British patients were evaluated. Most patients had branch RVO (52.95%), and 47.05% had central RVO. While anti-VEGF treatment significantly improved BCVA (*p* = 0.0061), Ozurdex did not result in additional gain (*p* = 0.747). Both treatments significantly reduced CRT (*p* = 0.0055 for anti-VEGF; *p* = 0.0079 for Ozurdex). No significant differences were observed between diabetic and non-diabetic BCVA patients receiving either treatment.

**Conclusion:**

Ozurdex emerges as a safe and effective option for persistent macular edema in RVO patients unresponsive to anti-VEGF therapy. Although structural improvements in CRT were observed following Ozurdex treatment, they did not correlate with additional gain in BCVA. Despite this, the reduced treatment frequency of Ozurdex compared to anti-VEGF injections may be advantageous, particularly for frail non-responding patients minimizing treatment burden.

## Introduction

Retinal vein occlusion (RVO) stands out as one of the most prevalent retinal vascular pathologies. It is marked by retinal haemorrhage, the emergence of new vessels, and the onset of macular edema, all of which culminate in diminished visual acuity. Typically, the severity of central edema correlates with the impairment of visual acuity in the majority of cases [1]. Alternatively, there are additional risk factors for ocular pathology such as diabetes mellitus, hypertension, or age-related macular edema [2]. Hypertension and other cardiovascular diseases were found in over 60% of the studied population according to Wykoff et al. [3]. According to Nicholson et al. [4], the most common risk factors for RVO are similar to the risk factors causing atherosclerosis and hyperviscosity status. The pathophysiology remains elusive, but the leading theory suggests vascular leakage due to vessel obstruction, leading to fluid accumulation and haemorrhage surrounding the retina, potentially resulting in ischemia. Furthermore, research indicates a post-retinal vein occlusion elevation in vascular endothelial growth factor levels [2, 5]. The diagnosis of RVO can be made clinically by observing retinal haemorrhage in conjunction with the tortuosity of retinal veins [6]. Retinal vein occlusion can lead to various complications, including retinal ischemia, optic neovascularization and neuropathy, retinal detachment, vitreous haemorrhage, and glaucoma [2, 7].

Treatment options for RVO typically entail intravitreal injections of either anti-VEGF agents or corticosteroids [8]. In the event of ischemia, vascular endothelial growth factor (VEGF) secretion may occur, exacerbating vascular leakage and retinal edema. As such, VEGF plays a pivotal role in the pathophysiology and symptoms of RVO [9]. Consequently, the goal of enhancing clinical outcomes for these patients has prompted the widespread adoption of intravitreal injections of anti-VEGF medications [10]. At the time of the study, the FDA and EMA had approved just two anti-VEGF drugs, ranibizumab and aflibercept, for the treatment of RVO. Bevacizumab, an additional VEGF inhibitor, is frequently utilised “off-label” in therapeutic settings. We acknowledge that Vabysmo has received approval from the FDA, EMA, and NICE.

The use of dexamethasone in the treatment of RVO hinges on its ability to halt the production of VEGF and inflammatory cells, which are crucial in the development of RVO. Additionally, dexamethasone can be utilized in other retinal vascular diseases such as diabetic retinopathy and retinal vasculitis [8, 11]. Several reported side effects of systemic steroid usage, including exacerbation of diabetes, osteoporosis, cushingoid state, adrenal suppression, glaucoma, and cataracts, should be taken into consideration [12].

The Dexamethasone Posterior Segment Drug Delivery System, known as Ozurdex (Allergan, Inc., Irvine, CA, USA), is a slow-release, intravitreal, biodegradable dexamethasone injectable implant. Upon intravitreal administration, the corticosteroid active medication, dexamethasone, bypasses the blood-retinal barrier, achieving a high intraocular concentration with minimal systemic absorption. It also exhibits anti-inflammatory and anti-VEGF properties. Enclosed within the implant, dexamethasone is gradually released due to a biodegradable copolymer matrix composed of lactic and glycolic acids [13]. Research has indicated that within the initial 2 months post-injection, there is a sustained presence of high levels of dexamethasone in the retina and vitreous. Furthermore, at lower concentrations, this presence can extend up to six months [14]. It is clinically utilized for the treatment of macular edema associated with RVO and non-infectious posterior uveitis, as well as macular edema related to retinitis pigmentosa, diabetic macular edema, and Irvine-Gass syndrome [15–18]. The intravitreal dexamethasone implant has demonstrated efficacy and received global approval for treating eyes with RVO. In an international 6-month study, the efficacy of a single 0.35 or 0.7 mg Ozurdex injection was compared with a sham injection [19].

## Methods

Notably, this study was conducted when Ozurdex was being approved as the second line to treat RVO now it is approved as the first line by the Royal College of Ophthalmologists. We conducted a retrospective observational cohort study to evaluate the structural and functional effects of Intravitreal implant (Ozurdex) treatment as a second-line option in patients with Retinal vein occlusion (RVO) and secondary macular edema who did not respond to first-line anti-VEGF therapy, either due to requiring frequent injections, experiencing poor visual outcomes, or showing no improvement in central retinal thickness (CRT). The study utilized data extracted from electronic health records of patients presenting with RVO between January 2014 and December 2019 at Salisbury District Hospital, Salisbury, UK. Inclusion criteria encompassed adult patients (age ≥ 60 years) with a confirmed diagnosis of either central retinal vein occlusion (CRVO) or branch retinal vein occlusion (BRVO) confirmed by ophthalmological specialists and surgeons. Patients with a history of any other retinal vascular diseases, prior ocular surgery, or inadequate follow-up data were excluded.

Each patient underwent a comprehensive ophthalmic assessment at baseline, which included measurement of central retinal thickness (CRT) and spectral-domain optical coherence tomography (SD-OCT) with best-corrected visual acuity (BCVA) evaluation. Demographic information, the duration of RVO, and the patient’s medical history were recorded. The primary outcome measure was the change in BCVA from baseline to various time points following Ozurdex treatment, along with the reduction in CRT measured by optical coherence tomography. Secondary outcomes included the requirement for additional interventions and any adverse events associated with Ozurdex treatment. Throughout the study period, strict adherence to patient confidentiality and privacy protocols was maintained.

Visual acuity was calculated using the logarithm of the minimum angle of resolution (logMAR) scale. Statistical analysis was conducted using IBM SPSS Statistics for Windows, Version 20.0 (IBM Corp., Armonk, NY, USA), employing repeated measures analysis of variance, paired-samples *t*-test, independent samples *t*-test, and Pearson’s correlation analysis. Statistical significance was defined as *p*-values less than 0.05.

## Results

Sixteen eyes of 16 patients, all of whom were White British, were retrospectively evaluated. The mean age of the entire study population was 75 ± 8.5 years (ranging from 62 to 90 years old). Thirty-two percent of patients were male (5 patients), and 68% were female (11 patients). Among all patients who experienced RVO, 47.05% had CRVO, and 52.95% had BRVO. None of the patients had undergone Laser/Intravitreal Triamcinolone Acetonide (IVTA) treatment. Regarding the vasculopathy status of the patients, many of the patients had one or more vascular risk factors. Nine patients had diabetes, of which only 1 had type I diabetes and 8 had type II diabetes. 11 patients had Hypertension and 6 patients had Ischaemic heart disease.

All patients underwent intravitreal anti-VEGF therapy initially, following which they were deemed non-responders to anti-VEGF treatment and subsequently received Ozurdex implant injections (700 µg). Most patients received the Ozurdex implants after several injections at short intervals with no improvement. Following the implant injection, none of the patients experienced operative complications. The mean baseline BCVA of patients prior to anti-VEGF injection therapy was 0.607 ± 0.365 logMAR, which improved to 0.297 ± 0.20 logMAR after anti-VEGF injections, demonstrating a statistically significant difference in the results (*p* = 0.0061). Conversely, Ozurdex treatment did not yield a statistically significant additional gain with a *p*-value of 0.747 and an average VA of 0.476 ± 0.33 logMAR (Fig. 1).

There was no statistically significant difference in VA between patients treated with anti-VEGF and Ozurdex implants (*p* = 0.0809). When comparing the effects of both injections on diabetic patients with RVO, anti-VEGF injections showed a statistically significant difference (*p* = 0.041), while Ozurdex injections showed no statistically significant difference (*p* = 0.852). However, there was no statistically significant difference between diabetic and non-diabetic patients receiving anti-VEGF and Ozurdex injections (*p* = 0.639 and *p* = 0.850, respectively).

The average baseline central retinal thickness (CRT) measurement was 505.18 ± 108.93 μm. Patients injected with anti-VEGF exhibited a mean CRT of 365.56 ± 151.61 μm, demonstrating a significant improvement in CRT measurements, with a statistically significant difference observed (*p* = 0.0055). Similarly, patients treated with Ozurdex implants showed an average CRT of 317.37 ± 94.74, which also displayed a statistically significant difference from baseline CRT measurements, with a *p*-value of 0.0079. However, there was no significant difference between anti-VEGF and Ozurdex implant injections in terms of CRT measurements (*p* = 0.289) (Fig. 2). Additionally, no statistically significant difference was reported in diabetic patients between treatment with anti-VEGF and Ozurdex implants (*p* = 0.144). The average number of Anti-VEGF injections was 20.5 ± 9.49 injection, compared to 2.13 ± 1.02 for Ozurdex implant. This difference was statistically significant (*p* < 0.0001), demonstrating that Ozurdex requires substantially fewer injections.

## Discussion

We conducted a retrospective evaluation to assess the efficacy of dexamethasone implants in patients with macular edema (ME) associated with retinal vein occlusion (RVO), aiming to ascertain the effectiveness of dexamethasone therapy as a secondary treatment option for individuals with poor or non-responsive outcomes to first-line treatment. Ozurdex, a potent steroid, was employed as an intravitreal dexamethasone implant to address ME associated with RVO. Consequently, it is plausible that both vascular changes and inflammation in RVO contribute to the development of ME [20].

In our study, patients treated with anti-VEGF injections exhibited a statistically significant difference between pre-treatment and post-treatment BCVA with a *p*-value of 0.0061. Conversely, Ozurdex treatment did not demonstrate a statistically significant additional improvement in BCVA, with a *p*-value of 0.747. observed that while Ozurdex injections led to a significant reduction in mean CRT in cases of macular edema associated with RVO, this improvement did not correlate with a mean improvement in BCVA. Similarly, Juniat et al. [21] demonstrated that patients treated with Ozurdex did not experience visual improvement, with a mean difference in VA post-treatment of − 0.08 logMAR (− 0.131 logMAR in our study). These findings may be attributed to the initial low baseline visual acuity and the presence of co-pathologies, both of which likely played a significant role in the absence of visual improvement. The lack of VA improvement following Ozurdex injections in cases of a dry macula is probably attributable to the same factors responsible for an inadequate response to anti-VEGF therapy[21]. Furthermore, the more severe cases of RVO, in which visual stability relied heavily on continued repetitive treatment, could be an influential factor [22].

Notably, Moisseiev et al. [23] emphasized that while Ozurdex has demonstrated efficacy in treating macular edema associated with both BRVO and CRVO, evidence from studies focusing on short-term efficacy (up to 12 months of follow-up) is limited to a maximum of two injections given at 6-month intervals. Such a timeframe may exceed the drug’s short-term effectiveness as demonstrated in previous studies [19, 24–26]. In the present study, although 53% of patients in this series received additional macular edema treatments after completing the GENEVA research regimen, none received regular treatment thereafter. Consequently, despite the impact of Ozurdex treatment, the final visual acuity closely mirrors the natural course of the baseline diagnosis, showing no significant change over a mean follow-up period exceeding 4 years [23]. Additionally, the authors noted that while there was a considerable reduction in retinal thickness, this did not correlate with final visual acuity. This variation between the BCVA and the CRT can be attributed to the retinal atrophy caused by the edema itself even after the clearing of macular edema.

On the contrary, Maggio et al. [27] documented a significant improvement in mean BCVA and CRT (*p* = 0.0001), with over 30% of eyes achieving an increase of ≥ 3 lines after 3 months of Ozurdex injections. The presence of foveal serous retinal detachment and macular ischemia was associated with poorer visual outcomes. The improvements observed were found to be closely associated with baseline BCVA and integrity of the ellipsoid zone [27]. Chiquet et al. [28] investigated the use of dexamethasone implants as an adjunct to anti-VEGF therapy for macular edema secondary to RVO. At the third month of treatment, the dexamethasone group exhibited superior visual recovery compared to the anti-VEGF group. However, no significant long-term optical or anatomical abnormalities were noted. Eris et al. [13] reported that a single dexamethasone implant led to a significant reduction in mean CRT in cases of macular edema associated with BRVO, with no significant adverse effects observed. However, this reduction did not correspond to an improvement in mean BCVA. The authors investigated patients with refractory macular edema due to BRVO, suggesting that steroids are beneficial in treating BRVO-related macular edema by reducing inflammation and stabilizing the blood-retinal barrier.

The effectiveness of the dexamethasone implant in patients previously treated with anti-VEGF therapy was demonstrated in a study by Tservakis et al. They reported that nine out of ten patients with retinal vein occlusion (RVO), who had shown inadequate response to anti-VEGF treatment, exhibited improved BCVA and reduced CRT following dexamethasone implant treatment [29]. This aligns with our findings regarding improvements in CRT, wherein the average CRT in patients injected with Ozurdex was 317.37 ± 94.74 μm, showing a statistically significant difference from baseline CRT measurements with a *p*-value of 0.0079. Castro-Navarro et al. [30] discussed that in the included 57-eyes, the baseline CRT was significantly decreased from 567.6 ± 226.2 to 326.9 ± 141.0 μm with a *p*-value of < 0.0001. Likewise, Li et al. [31] documented that dexamethasone demonstrated superiority in anatomic outcomes as the mean CRT reduction from baseline was significantly larger in the dexamethasone group compared with other groups (*p* < 0.001) [31].

In the SOLO retrospective 6-month analysis, dexamethasone implant therapy was associated with greater improvement in CRT in BRVO of shorter duration [32]. Additionally, in the GENEVA pivotal randomized clinical trials evaluating dexamethasone implant for the treatment of ME related to RVO, a shorter duration of ME was linked to higher improvements in BCVA and CRT following dexamethasone implant treatment [33]. Although a re-treatment frequency of 4–5 months may be necessary for optimal efficacy, dexamethasone therapy requires far fewer intravitreal injections compared to anti-VEGF therapy. Thus, dexamethasone therapy for RVO-related ME offers the advantage of reducing the treatment burden associated with intravitreal injections [31].

This study demonstrates that Ozurdex maintains a favourable safety profile for the treatment of ME associated with RVO, consistent with previous findings [24, 34]. Any increases in IOP were generally managed with topical medications, and none of the patients in the dexamethasone-treated group required incisional glaucoma surgery. The mean elevation in IOP following retreatment with dexamethasone was comparable to that observed after initial therapy. These findings align with those of the three-year MEAD study investigating dexamethasone implant treatment in patients with DME, which revealed no cumulative effect of successive implants on IOP and no increase in the incidence of IOP elevation following repeat treatment [35].

Our retrospective observational cohort study aimed to assess the structural and functional effects of Intravitreal implant (Ozurdex) treatment as a second-line option for patients with RVO and secondary macular edema who did not respond to first-line anti-VEGF therapy. The study design featured clear objectives, comprehensive data collection, a substantial sample size of 16 eyes from 16 patients, appropriate statistical analysis methods, and a clear presentation of results. However, the small number of participants and the retrospective nature of this study limited its scope, potentially impacting the generalizability of the findings. Another limitation is that the anti-VEGF group underwent multiple consecutive injections, while the dexamethasone group received fewer injections, introducing variability in treatment regimens. Despite these limitations, the study benefitted from uniformity in procedures performed by the same specialists for all patients, enhancing internal validity. Nonetheless, these factors should be considered when interpreting the results and extrapolating conclusions.

## Conclusion

In conclusion, our study underscores the safety and potential benefits of Ozurdex as a treatment option for persistent ME resulting from RVO in patients who exhibit poor or incomplete responses following at least three consecutive monthly intravitreal anti-VEGF injections. The complex molecular mechanisms underlying ME development and the impact of corticosteroids on these mechanisms support the efficacy of Ozurdex in such cases. Interestingly, diabetic and cardiovascular disease-related RVO patients displayed distinct responses compared to non-diabetic counterparts, with a higher proportion of non-responders to anti-VEGF therapy among diabetics and cardiovascular disease patients. This observation suggests that general vasculopathy pathophysiology may influence the prognosis of RVO. Our findings revealed that while structural improvements, as indicated by the CRT parameter, were observed after Ozurdex implantation, these improvements did not translate into additional improvements in BCVA. Despite the lack of additional significant BCVA improvement with Ozurdex, its reduced treatment frequency compared to anti-VEGF injections may be advantageous, particularly considering the potential frailty of non-responding patients. Thus, Ozurdex represents a valuable alternative for managing ME associated with RVO, offering a balance between efficacy and treatment burden in these challenging cases.
